# New Structure of *Aeromonas salmonicida* O-Polysaccharide Isolated from Ill Farmed Fish

**DOI:** 10.3390/microorganisms12081575

**Published:** 2024-08-01

**Authors:** Karolina Ucieklak, Sylwia Wojtys-Tekiel, Garance Leroy, Laëtitia Le Devendec, Sandrine Baron, Marta Kaszowska

**Affiliations:** 1 Laboratory of Microbial Immunochemistry and Vaccines, Ludwik Hirszfeld Institute of Immunology and Experimental Therapy, Polish Academy of Sciences, 53-114 Wroclaw, Poland; karolina.ucieklak@hirszfeld.pl (K.U.); sylwia.wojtys-tekiel@hirszfeld.pl (S.W.-T.); 2 Laboratory of Marine Biotechnology and Chemistry, University of Western Brittany, EMR CNRS 6076, IUEM, 29000 Quimper, France; 3 Mycoplasmology-Bacteriology and Antimicrobial Resistance Unit Ploufragan Plouzane-Niort Laboratory (ANSES), 22440 Ploufragan, France; laetitia.ledevendec@anses.fr (L.L.D.);

**Keywords:** *Aeromonas salmonicida*, O-polysaccharide diversity, HR MAS NMR, lipopolysaccharide

## Abstract

The diversity of O-polysaccharides (O-antigens) among 28 *Aeromonas salmonicida* strains isolated from ill fish has been determined by using high-resolution magic angle spinning (HR MAS) NMR spectroscopy. The new O-polysaccharide has been identified in two isolates. This new structure was investigated by ^1^H and ^13^C NMR spectroscopy and matrix-assisted laser-desorption/ionization time-of-flight mass spectrometry (MALDI-TOF MS). The following structure of the linear hexasaccharide repeating unit of *A. salmonicida* O-antigen has been established: →3)-α-L-Rha*p*-(1→3)-α-D-Man*p*NAc-(1→2)-β-D-Glc*p*-(1→3)-α-L-Rha*p*2OAc4OAc-(1→3)-β-D-Man*p*NAc-(1→3)-α-D-Glc*p*-(1→. This new *A. salmonicida* O-polysaccharide was detected among two isolates collected from trout and turbot fish in 2010 and 2011, respectively. Further investigations should be conducted to evaluate the distribution of this new O-polysaccharide among a larger collection of isolates, depending on their geographic origin, the species of fish, and the health status of the fish.

## 1. Introduction

*Aeromonas salmonicida* is an important fish pathogen, causing the systemic disease furunculosis in a great variety of fish (e.g., salmon, trout, and turbot). Since the annual worldwide losses of farmed fish due to diseases involve millions of dollars, this pathogen has been subjected to considerable investigation [1]. One of the principal virulence factors of this pathogen is an S-layer (named the A-layer) that consists principally of a 2-dimensional crystalline tetragonal protein (A-protein, with a molecular mass of 49 kDa) array, which is tethered to the cell by lipopolysaccharide (LPS) molecules. Labeling studies have shown that the A-layer appears to cover most of the surface of virulent *A. salmonicida*, although some parts of LPS (O-polysaccharide) may also be exposed. This structure has been shown to protect this bacterium from killing by serum in a manner that somehow requires both LPS and the A-layer [2].

LPS is an amphiphilic molecule which is a well-characterized pathogen-associated molecular pattern (PAMP). It is a powerful activator of innate immune responses. The characteristics of endotoxin are important, since the physiological and pathophysiological effects depend strongly on their chemical structure. It consists of three domains: lipid A, core oligosaccharide, and O-specific polysaccharide (O-antigen, O-serotype). By now, only one structure of *A. salmonicida* lipid A, one core oligosaccharide, and three O-polysaccharides have been identified and published [3,4,5,6,7,8].

Antibiotherapy is still used against furonculosis and has led to resistance [9]. Several studies have reported the detection of resistant *A. salmonicida* strains in trout fish farms in Denmark [10], France [11], Turkey [12], and Canada [13]. Colistin is a positively charged peptide that exhibits activity against Gram-negative bacteria by interacting with its lipopolysaccharide (LPS) molecules. These interactions involve the electrostatic attraction between the positively charged diaminobutyric acid (Dab) residue of colistin and the negatively charged phosphate groups present in the lipid A part of the bacterial membrane. As a result of this interaction, the LPS becomes destabilized, leading to an increase in the permeability of the bacterial membrane inducing cell lysis and death. This mode of action is common to all polymyxins, including polymyxin B [14].

Herein, a structural study of O-polysaccharide diversity among a collection of 28 *A. salmonicida* strains collected from diseased farmed fish, including trout and turbot, in France has been established.

## 2. Materials and Methods

### 2.1. Bacterial Strains

#### 2.1.1. Origin and Identification

The bacterial collection is composed of 28 strains of *A. salmonicida* isolated from diseased fish between 1994 and 2014. The isolates were collected from rainbow trout (n = 9) and turbot (n = 19) bred in 13 fish farms. All isolates were provided by French laboratories located in western France, the Mycoplasmology-Bacteriology and Antimicrobial resistance unit (MBA) and Virology, Immunology and Ecotoxicology of Fish unit (VIMEP) of the Ploufragan-Plouzané-Niort Laboratory of the French Agency for Food, Environmental and Occupational Health & Safety (Anses, Ploufragan, France), and Labocéa (site of Quimper). Strains had been preserved by freezing at −70 °C. All strains were purified on tryptic soy agar and identified at genus level by MALDI-TOF Biotyper platform (Bruker, Bremen, Germany) in Labocéa laboratory. In MBA unit, the identification at species level was confirmed by sequencing the housekeeping gene *gyrB* [15,16], and the determination of the subspecies *A. salmonicida* subsp *salmonicida,* using PCR, targeted the phage PSal 3, which is specific to the subspecies *salmonicida* [15].

#### 2.1.2. Genetic Diversity

Strain genotyping was performed using the enterobacterial repetitive intergenic consensus PCR (*ERIC-PCR*) typing method. The amplification conditions were as follows: initial denaturation at 95 °C for 5 min, followed by 35 amplification cycles that successively comprised denaturation at 92 °C for 45 s, annealing at 52 °C for 1 min, and extension at 65 °C for 4 min, before a final extension step at 70 °C for 20 min [17].

#### 2.1.3. Antimicrobial Susceptibility Testing

The minimal inhibitory concentration (MIC) was determined using the broth microdilution method. Five antibiotics are included in the custom-made microdilution plate “FRANV” (Thermofischer, Dardilly, France): colistin (COL) (0.5–32.0 µg/L), florfenicol (FLO) (0.12–16.0 µg/L), oxolinic acid (OXO) (0.004–8.0 µg/L), oxytetracycline (OXY) (0.03–16.0 µg/L), and trimethoprim-sulfamethoxazole (SXT) (0.015/0.3–8.0/152.0 µg/L). Excluding colistin, the four other antibiotics are labelled for aquaculture in France. The colistin was tested because it belongs to the polymyxin class, which is known to interact with LPS molecules. Results were recorded after a 48 h incubation at 22 °C using the testing protocols recommended for non-fastidious microorganisms in the guidelines proposed by the Clinical Laboratory Standard Institute guideline (CLSI. Methods for antimicrobial broth dilution and disk diffusion susceptibility testing of bacteria isolated from aquatic animals, 2nd ed. CLSI Guideline Vet 03. Wayne, PA: Clinical and Laboratory Standards Institute, 2020).

Interpretation criteria are epidemiological cut-off values (Ecoff). The Ecoff values allow categorizing an isolate as wild-type (WT) or non-wild-type (NWT). A microorganism is defined as WT for a species by the absence of acquired and mutational resistance mechanisms to the drug in question. A microorganism is categorized as WT for a species by applying the appropriate cut-off value in a defined phenotypic test system [18].

The Ecoff proposed by CLSL VET 04 (CLSI. *Performance Standards for Antimicrobial Susceptibility Testing of Bacteria Isolated From Aquatic Animals* 3rd ed. CLSI Supplement VET 04 Wayne, PA: Clinical and Laboratory Standards Institute, 2020) was used for oxolinic acid (WT ≤ 0.12, NWT ≥ 0.25), oxytetracycline (WT ≤ 1.0, NWT ≥ 2.0), florfenicol (WT ≤ 4.0, NWT ≥ 8.0). For colistin (WT ≤ 4.0, NWT ≥ 8.0) and trimethoprim-sulfamethoxazole (WT ≤ 0.25/4.75, NWT ≥ 0.5/9.5), Ecoff was not available in CLSI, so the one previously proposed by Baron et al., 2017 [19] was used (Table 1).

### 2.2. Isolation of the Lipopolysaccharide and O-Polysaccharide

Bacteria were cultivated aerobically with aeration at 22 °C for 24 h, in peptone water, then harvested and freeze-dried. The LPS was extracted from bacterial cells by the hot phenol/water method [20], purified from nucleic acids by ultracentrifugation (105,000× *g*, 4 °C, 3 × 6 h) with a yield of 1.4%. *A. salmonicida* 11/A/658 LPS (200 mg) was degraded by 1.0% acetic acid at 100 °C for 100 min. The supernatant was fractionated using the semi-preparative HPLC UltiMate 3000 chromatographic system (Dionex Corporation, Sunnyvale, CA, USA) on a HiLoad 16/600 Superdex 30 prep grade column (30 mm × 124 cm, grain size 34 μm, GE Healthcare, Chicago, IL, USA) equilibrated with 0.05 M acetic acid. Eluates were monitored with a Shodex RI-102 detector (Showa-Denko, Tokio, Japan). All fractions were checked by MALDI-TOF MS. The polysaccharide fraction was subjected to further structural analysis by NMR spectroscopy.

#### Instrumental Methods

NMR Spectroscopy. NMR spectra were recorded using a Bruker Avance III 600 MHz spectrometer (Bruker Biospin GmbH, Rheinstetten, Germany) using a 5 mm QCI ^1^H/^13^C/^15^N/^31^P probe equipped with a z-gradient. The measurements were performed at 298 K. The O-specific polysaccharide was dissolved in ^2^H_2_O and the acetone (δ_H_/δ_C_ 2.225/31.05 ppm) was used as an internal reference. The data were acquired and processed using Bruker Topspin software (version 3.1) and with the help of the NMRFAM-SPARKY program [21]. The signals were assigned by one-dimensional (^1^H, ^13^C, ^31^P) and two-dimensional (2D) experiments: correlation spectroscopy (COSY), total correlation spectroscopy (TOCSY, with mixing times: 30, 60, and 100 ms), nuclear Overhauser effect spectroscopy (NOESY, with mixing time of 100 ms), ^1^H-detected heteronuclear single quantum coherence spectroscopy (HSQC) with and without carbon decoupling, HSQC-TOCSY, and ^1^H-^13^C heteronuclear multiple-bond correlation spectroscopy (HMBC, with mixing time of 60 ms) [22,23].

HR MAS NMR Spectroscopy. The HR MAS NMR was used to create a database of *A. salmonicida* O-serotypes (O-polysaccharides). NMR spectra were obtained using a ^1^H, ^13^C high-resolution magic angle spinning (HR MAS) probe with *z*-gradients at the magic angle. LPS (3–4 mg) were suspended in ^2^H_2_O and placed into the ZrO_2_ rotor. HR MAS NMR experiments were carried out at a spin rate of 4 kHz at 30 °C (the measured temperature of the bearing air used for sample spinning) [24].

Mass Spectrometry. O-polysaccharide (1 mg/mL in mQ) fraction was analyzed using a MALDI-TOF Ultraflextreme III instrument (Bruker Daltonic GmbH, Bremen, Germany). The MALDI-TOF MS spectra were obtained in a positive ion mode. For analyses, 2,5-Dihydroxybenzoic acid (10 mg/mL in 1:1 AcN/0.2 M citric acid (*v*/*v*)) was used as a matrix.

## 3. Results

### 3.1. A. salmonicida O-Polysaccharides Database

**Figure 1:**
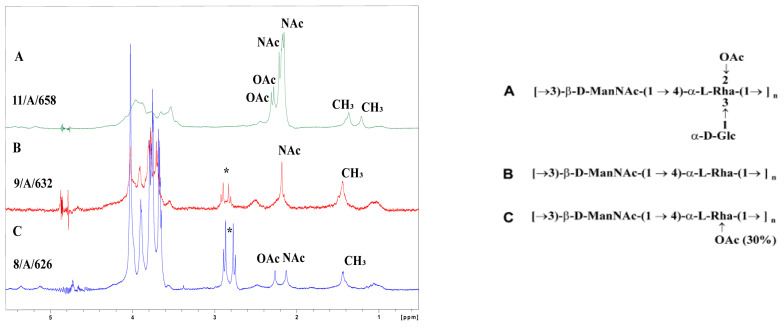
^1^H HR MAS NMR spectra of *A. salmonicida* O-polysaccharides: (A) strain 11/A/658 with the new O-polysaccharide structure, (B) strain 9/A/632 with the structure B, and (C) strain 8/A/626 with the structure A. Chart represents A, B, C structures of *A. salmonicida* O-polysaccharides which have been identified [6]. * impurities from the microextraction step.

Up to this time three different structures of *A. salmonicida* O-polysaccharides have been identified (chart in Figure 1) [6].

Herein, by using ^1^H and ^1^H-^13^C HR MAS NMR, a database of 28 *A. salmonicida* O-polysaccharide structures has been created. The trisaccharide structure A was identified in 23 *A. salmonicida* strains based on the presence of a characteristic signal from a CH_3_ group (→4)-α-L-Rha*p* residue), and signals belonging to NAc (of →3-β-D-Man*p*NAc residue) and OAc groups. The spin systems of these residue/groups with the addition of terminal α-D-Glc*p* residue have been proven. The disaccharide structure B has been determined in three strains by the presence of signals of →3-β-D-Man*p*NAc and→4)-α-L-Rha*p* residue only. No strain with a known C structure has been isolated. Additionally, in two *A. salmonicida* strains 11/A/658 (Figure 1) and 10/A/646, a new O-polysaccharide structure has been identified based on characteristic signals belonging to two Rha*p* residues (CH_3_), and two NAc and two OAc groups (Table 2). The strain 11/A/658 has been selected for further structural analysis.

#### 3.1.1. Isolation of the O-Polysaccharide

LPS of *A. salmonicida* 11/A/658 was isolated from bacterial mass. The mild acid hydrolysis of the LPS yielded five polysaccharide (PS) and oligosaccharide (OS) fractions: PSI-III consisting of a core oligosaccharide substituted by several repeating units, and OSIV-V—the unsubstituted core oligosaccharide fractions. The high yield of PSI-III suggested the smooth (S-LPS) type of *A. salmonicida* 11/A/658 LPS. The data presented herein concern the PSI fraction.

#### 3.1.2. Structural Analysis

**Figure 2:**
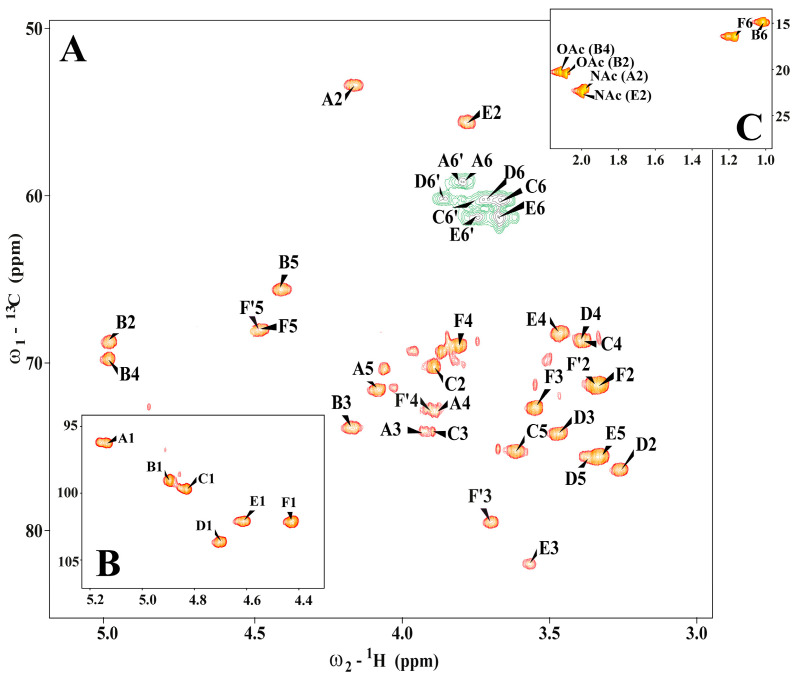
(**A**–**C**) Selected regions of ^1^*J*_H,C_- and ^3^*J*_H,C_-connectivities in ^1^H-^13^C HSQC-DEPT spectrum of the *A. salmonicida* 11/A/658 O-polysaccharide. The cross-peaks are marked as uppercase letters indicated in the text.

The initial ^1^H NMR investigation of the *A. salmonicida* 11/A/658 O-polysaccharide indicated the presence of two N-acetyl (NAc), two O-acetyl (OAc) groups, and two deoxy sugar residues (the presence of CH_3_ groups). The ^1^H NMR spectrum of the *A. salmonicida* 11/A/658 contained the six signals of anomeric protons. The ^1^H-^1^H COSY and ^1^H-^1^H TOCSY (using different mixing times) allowed for the assignment of the H-1 to H-6,6′ signals for each residue, whereas the ^1^H-^13^C HSQC-DEPT spectrum also contained signals for carbons (Table 2, Figure 2).

Residue A, with H-1/C-1 signals at δ_H_/δ_C_ 5.14/96.2 ppm (^1^*J*_C-1,H-1_ = 173 Hz), was recognized as the 3-substituted α-D-Man*p*NAc residue based on the chemical shift of the C-2 (δ_C_ 53.6 ppm) and C-3 (δ_C_ 74.1 ppm). The chemical shift was inferred by comparison with published data [24]. Residue B, with H-1/C-1 signals at δ_H_/δ_C_ 4.90/98.9 ppm (^1^*J*_C-1,H-1_ = 173 Hz), was recognized as 3-substituted α-L-Rha*p*2OAc4OAc on the basis of the signals of exocyclic CH_3_ groups (δ_H_/δ_C_ 1.02/14.9 ppm) and the small vicinal coupling constants between H-1 and H-2. Relative downfield chemical shifts of the C-3 at δ_C_ 74.0 ppm indicated a substitution position. Characteristic chemical shift values of H-2/C-2 (δ_H_/δ_C_ 5.00/68.9 ppm) and H-4/C-4 (δ_H_/δ_C_ 5.00/69.9 ppm) indicated substitution by two OAc groups (δ_H_/δ_C_ 2.08/20.6, δ_C_ 173.2 ppm and δ_H_/δ_C_ 2.11/20.2, δ_C_ 173.3 ppm, respectively). Residue C, with H-1/C-1 signals at δ_H_/δ_C_ 4.83/99.7 ppm (^1^*J*_C-1,H-1_ = 176 Hz), was recognized as 3-substituted α-d-Glc*p* based on the characteristic five-proton spin systems. Significant downfield shift was observed for C-3 of this residue (δ_C_ 74.3 ppm) as compared with the chemical shift of the corresponding non-substituted monosaccharide, indicating the linkage position for this residue. Residue D, with H-1/C-1 signals at δ_H_/δ_C_ 4.70/103.6 ppm (^1^*J*_C-1,H-1_ = 162 Hz), was recognized as 2-substituted β-d-Glc*p* based on the characteristic chemical shift of the C-2 (δ_C_ 76.4 ppm), the similarity of the ^1^H and ^13^C chemical shifts with those of β-d-Glc*p,* and the large vicinal couplings between all protons in the sugar ring. Residue E, with H-1/C-1 signals at δ_H_/δ_C_ 4.61/102.1 ppm (^1^*J*_C-1,H-1_ = 163 Hz), was recognized as 3-substituted β-d-Man*p*NAc based on the characteristic five-proton spin systems, chemical shift value of the C-2 (δ_C_ 55.6 ppm), and high ^13^C chemical shift of the C-3 (δ_C_ 82.0 ppm). Residue F, with H-1/C-1 signals at δ_H_/δ_C_ 4.43/102.3 ppm (^1^*J*_C-1,H-1_ = 162 Hz), was recognized as terminal β-L-Rha*p* based on the characteristic spin system and the signals of the CH_3_ group (δ_H_/δ_C_ 1.20/16.5 ppm). Additionally, also the signals for 3-substituted β-L-Rha*p* (described as F’ residue) have been identified based on the characteristic chemical shift of the C-3 (δ_C_ 79.5 ppm) and for the CH_3_ group (δ_H_/δ_C_ 1.17/16.4 ppm). The presence of terminal and 3-substituted residue points for fraction heterogeneity related to the presence mixture of different lengths of linear *A. salmonicida* O-polysaccharides. 

**Figure 3:**
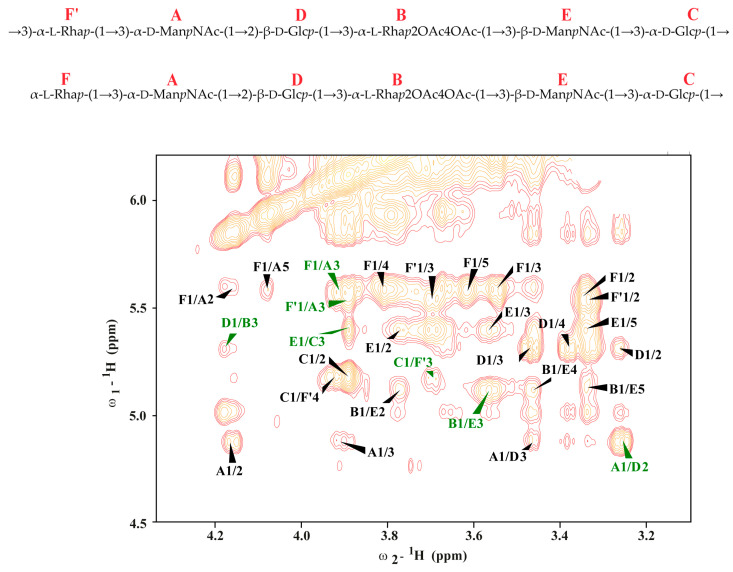
Selected region of the ^1^H-^1^H NOESY spectrum of the *A. salmonicida* 11/A/658 O-polysaccharide (PSI fraction).

The monosaccharide sequence was established using ^1^H-^1^H NOESY experiment. The ^1^H-^1^H NOESY spectrum showed strong inter-residue cross-peaks between the following transglycosidic protons: H-1 of **B**/H-3 of **E**, H-1 of **D**/H-3 of B, H-1 of **E**/H-3 of **C**, H-1 of **F**/**F’**/H-3 of A, H-1 of **C**/H-3 of **F’**, and H-1 of **A**/H-2 of **D** (Figure 3). The results suggest the following structure of the repeating unit of the A*. salmonicida* 11/A/658 O-polysaccharide.

**Figure 4:**
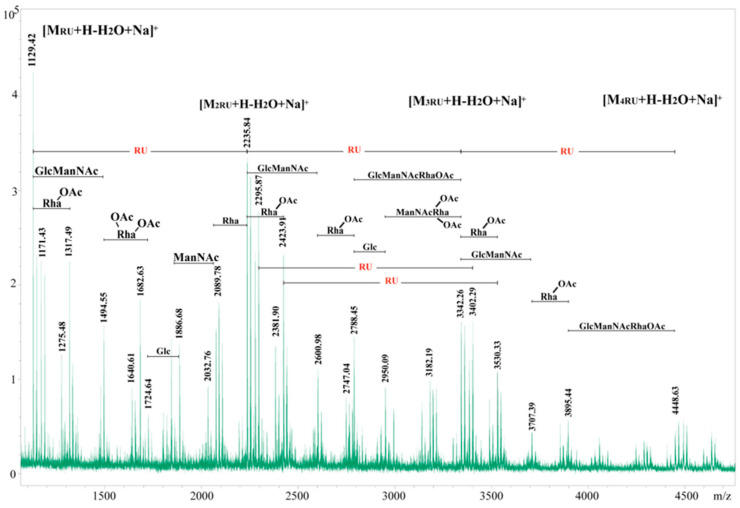
MALDI-TOF MS spectrum of the *A. salmonicida* 11/A/658 O-polysaccharide (PSI fraction).

The structure of the repeating unit of *A. salmonicida* O-polysaccharide 11/A/658 was analyzed by MALDI-TOF MS (Figure 4). The six sugar residues, two Man*p*NAc, two Glc*p*, and two Rha*p* residues, give together a monoisotopic mass of 1106.401 Da (**M_RU_**). The ions at *m*/*z* 1129.42 Da [M_RU_ + H-H_2_O + Na]^+^, at *m*/*z* 2235.84 [M_2RU_ + H-H_2_O + Na]^+^, at *m*/*z* 3342.26 [M_3RU_ + H-H_2_O + Na]^+^, and at *m*/*z* 4448.63 [M_4RU_ + H-H_2_O + Na]^+^ represent the masses of one, two, three, and four complete repeating units, respectively.

#### 3.1.3. Origin, Genetic Diversity, and Antimicrobial Susceptibility of Bacterial Strains and *A. salmonicida* O-Polysaccharide Structures

The 28 isolates included in this study were collected from turbot (n = 18) and from trout (n = 10), bred in five and nine farms, respectively. Twenty-six out of the twenty-eight A. salmonicida isolates had O-polysaccharide structures, which have been previously described (Figure 2), structure A (n = 18) or structure B (n = 8). The two isolates, which have the new O-polysaccharide structure (new A. salmonicida O-serotype), were collected in 2010 from a trout and in 2011 from a turbot (Table 1).

The three O-polysaccharide structures were found in both species of fish. Out of the 18 isolates with structure A, 7 were collected from trout and 11 were collected from turbot. Structure A was the dominant in both species of fish. No link between LPS structure and the origin of the strain (fish species) was observed.

Among the thirteen isolates collected in turbot farm 7, three LPS structures, eight A-type structures, four B-type structures, and one new structure were observed.

The genetic diversity of these 28 *A. salmonicida* strains was investigated by ERIC-PCR. The strains are distributed into fifteen genetic profiles (called ERIC-profiles). The eighteen strains with structure A are distributed among eleven ERIC-profiles and the eight strains with structure B into seven ERIC-profiles. Six ERIC-profiles are composed of 2 or more strains. Five of them are composed of strains with structure A and structure B. No link between the ERIC-profiles and the LPS (O-polysaccharide structures) was observed, meaning that these three structures could be detected in a large diversity of A. salmonicida strains.

The antimicrobial susceptibility was tested using a microbroth dilution for five antibiotics, colistin and the four others labelled for use in aquaculture.

No strain was wild-type for the four antibiotics labeled for use in aquaculture in France (oxolinic acid (OXO), oxytetracylcine (OXY), florfenicol (FLO), and trimethoprim-sulfamethoxazole (SXT)). In contrast, two strains were non-wild-type for the four antibiotics. They both have an O-polysaccharide A structure. Thirteen strains (eight structure A, four structure B, and one new structure) were non-wild-type for three agents, “oxy-oxo-sxt”. Multidrug resistant isolates are defined as isolates that are not susceptible to at least one agent in at least three antimicrobial classes [25,26]. By extension of this definition, fifteen strains of this study (53.6%) are multi-drug “non wild type”. Strains with structure A and strains with structure B are both present in all different antimicrobial profiles, except the non-wild-type profile for the four antibiotics. 

One of the strains with the new structure is a non-wild-type only for oxolonic acid and a second one is a multi-drug non-wild-type. Based on the Ecoff proposed by Baron et al. [19], all the isolates were wild-type to colistin independently of the O-polysaccharide structures.

## 4. Discussion

*A. salmonicida* is an important pathogen for fish, especially salmonidae, which is the causative agent of furonculosis. Antibiotic treatment is not always efficient due to the acquisition of resistance by the strain. Moreover, a reduction in the usage of antibiotics is encouraged by most international organizations. One of the alternatives is phagotherapy. The lipid A of the LPS and A-layer have been identified as a receptor for *Aeromonas subsp salmonicida* myophages [27,28]. The determination of an LPS with new O-polysaccharide structures (new O-serotypes) could impact the efficiency of phagotherapy.

Moreover, the A-layer is known as one of the *A. salmonicida* major virulence factors [29]; the O-polysaccharide (as a part of LPS) may modulate this virulence by direct presence between tetragonal A-protein arrays.

In this study, a new *A. salmonicida* O-polysaccharide has been identified in two clinical isolates collected from trout and turbot. Despite the different O-polysaccharide structures being currently dominant among the tested isolates (structure A in Figure 2), this may change in the future.

The HR MAS NMR is dedicated for fast LPS profile determination. The creation of a useful *A. salmonicida* O-polysaccharide database will be useful in the future for the rapid identification of different O-serotypes in new isolates (available for European veterinarian laboratories). Collaboration with many different fish farms (European veterinarian laboratories) gives the possibility of structural identification, which is also important for the control of diversity among *A. salmonicida* isolates.

The investigation of the O-polysaccharide structures presented on *A. salmonicida* isolates could bring information about or could be linked with other traits of *Aeromonas*, including geographical diversity (by using strains isolated from many different European fish farms). Such data could be used to improve the identification at species level, and the understanding of *A. salmonicida* pathogenicity. The results related to the O-polysaccharide structure presented in isolates can be used as one of the necessary steps to propose alternative methods of control of *A. salmonicida* infections and can be used to develop a rapid, accurate detection system in fish farming. Moreover, Hofer et al. recently explored the ability of non-A-layer and A-layer *A. salmonicida* strains to incorporate polyunsaturated fatty acids (PUFAs) into their lipid profiles and tested the phenotypic effects thereof. Temperature-dependent effects on biofilm formation were observed [30]. This study compared strains with and without the A-layer, but not between the A-layer strain and different O-polysaccharide structures. Further investigations are needed to understand the impact of the diversity on the O-polysaccharide structure and thus on biofilm formation.

However, studying the structure of LPS in pathogenic bacteria is crucial to assist in the search for effective compounds in combating antibiotic resistance. Indeed, antibiotics under development, such as antimicrobial peptides, interact with the LPS of Gram-negative bacteria. For example, alterins or ogipeptins are cationic antimicrobial peptides which are produced by *Pseudoalteromonas* strains and exert their antimicrobial action by interacting with the LPS. Enhancing our comprehension of the LPS structure could provide insights into a more accurate mode of action, facilitating the targeting of Gram-negative pathogens [14,31,32,33].

Based on these preliminary observations, the LPS structure seems to not be linked to a particular species. The new structure has been found in trout farms and turbot farms; this could mean that this new structure, as with the structures A and B, is not specific to fish species. Concerning the antimicrobial profile and ERIC-profile, they are not associated with a specific O-polysaccharide structure. All the isolates included in this study were wild-type for colistin and differences were observed even in MIC concentration. Nevertheless, the number of strains with the new O-polysaccharide structure are too few to allowed definitive conclusion. Several questions are pending. (I) What is the proportion of the new structure inside A. salmonicida subs salmonicida, and in other subspecies of *A. salmonicida*? (II) Does this new structure impact the bacteria–phage interaction? (III) Does the A. salmonicida O-polysaccharide structure confer a protection against macrophage?

## 5. Conclusions

Identification of *A. salmonicida* O-polysaccharides (different O-serotypes) presented in ill fish could be very useful for a veterinarian either in confirming the etiologic agent of disease or in improving biosecurity of fish farms, by providing a quick tool to detect the presence of pathogenic *A. salmonicida* O-polysaccharides before the level of the pathogen reaches density and causes disease. The structural identification of *A. salmonicida* O-polysaccharides could contribute to reducing and improving the usage of antibiotics in fish farms, which are considered a potential hotspot for antimicrobial resistance dissemination.

## Figures and Tables

**Table 1:** Repartition of the 28 structures of *A. salmonicida* O-polysaccharides depends on the origin of the strain (date of isolation and fish species) and the antimicrobial profile.

STRAIN	YEAR	SPECIES	FISH-FARM	ERIC-PCR PROFILE	OXY	OXO	SXT	FLO	COL	AMR PROFILE	O-Polysaccharide Type
13/A/730	2013	trout	farm 32	P9	WT	NWT	WT	WT	WT	oxo	A
10/A/646	2010	trout	farm 34	P9	WT	NWT	WT	WT	WT	oxo	NEW
A/94/20	1994	turbot	farm 18	P4	WT	NWT	NWT	WT	WT	oxo-sxt	A
A/96/205	1996	trout	farm 0	P25	NWT	WT	NWT	WT	WT	oxy-sxt	A
A/96/21	1996	turbot	farm 7	P17	NWT	WT	NWT	WT	WT	oxy-sxt	A
9/A/635	2009	trout	farm 27	P9	WT	NWT	NWT	WT	WT	oxo-sxt	A
11/A/652	2011	turbot	farm 11	P21	WT	NWT	NWT	WT	WT	oxo-sxt	A
13/A/734	2013	turbot	farm 7	P17	WT	NWT	NWT	WT	WT	oxo-sxt	A
A/96/22	1996	turbot	farm 7	P16	NWT	WT	NWT	WT	WT	oxy-sxt	B
9/A/640	2009	trout	farm 25	P4	WT	NWT	NWT	WT	WT	oxo-sxt	B
9/A/629	2009	turbot	farm 7	P14	NWT	NWT	NWT	WT	WT	oxy-sxt	B
9/A/632	2009	turbot	Farm 13	P21	WT	NWT	NWT	WT	WT	oxo-sxt	B
9/A/638 *	2009	turbot	farm 13	P24	WT	NWT	NWT	WT	WT	oxo-sxt	B
A/94/18	1994	trout	farm 6	P2	NWT	NWT	NWT	WT	WT	oxo-oxy-sxt	A
A/98/43	1998	trout	farm 8	P2	NWT	NWT	NWT	WT	WT	oxo-oxy-sxt	A
7/A/621	2007	turbot	farm 7	P12	NWT	NWT	NWT	WT	WT	oxo-oxy-sxt	A
8/A/626	2008	trout	farm 9	P6	WT	NWT	WT	WT	WT	oxo	A
10/A/641	2010	turbot	farm 7	P15	NWT	NWT	NWT	WT	WT	oxo-oxy-sxt	A
10/A/644	2010	turbot	farm 7	P16	NWT	NWT	NWT	WT	WT	oxo-oxy-sxt	A
11/A/666	2011	turbot	farm 7	P16	NWT	NWT	NWT	WT	WT	oxo-oxy-sxt	A
11/A/667	2011	turbot	farm 7	P19	NWT	NWT	NWT	WT	WT	oxo-oxy-sxt	A
12/A/688	2012	trout	farm 19	P4	NWT	NWT	NWT	WT	WT	oxo-oxy-sxt	A
10/A/651	2010	turbot	farm 7	P16	NWT	NWT	NWT	WT	WT	oxo-oxy-sxt	B
11/A/656	2011	trout	farm 25	P5	NWT	NWT	NWT	WT	WT	oxo-oxy-sxt	B
11/A/657	2011	turbot	farm 7	P17	NWT	NWT	NWT	WT	WT	oxo-oxy-sxt	B
11/A/658	2011	turbot	farm 7	P18	NWT	NWT	NWT	WT	WT	oxo-oxy-sxt	NEW
10/A/649	2010	turbot	farm 7	P17	NWT	NWT	NWT	NWT	WT	oxo-oxy-sxt-flo	A
14/A/756	2014	turbot	farm 12	P21	NWT	NWT	NWT	NWT	WT	oxo-oxy-sxt-flo	A

* One strain did not belong to the subspecies salmonicida; it was collected from turbot in 2009.

**Table 2:** ^1^H and ^13^C NMR chemical shifts of *A. salmonicida* 11/A/658 O-polysaccharide.

	Chemical Shifts (ppm)						
Sugar Residue	H1/C1	H2/C2	H3/C3	H4/C4	H5/C5	H6, H6′/C6	NAc/OAc
**A** →3)-α-D-Man*p*NAc-(1→	5.14 96.2	4.16 53.6	3.93 74.1	3.90 72.9	4.08 71.6	3.79, 3.81 59.1	1.98 175.1
**B** →3-α-L-Rha*p*2OAc4OAc-(1→	4.90 98.9	5.00 68.9	4.17 74.0	5.00 69.9	4.42 65.7	1.02 14.9	2.08/2.11 173.2/173.3
**C** →3)-α-D-Glc*p*-(1→	4.83 99.7	3.89 70.3	3.89 74.3	3.39 68.7	3.62 75.2	3.67, 3.79 60.3	
**D** →2)-β-D-Glc*p*-(1→	4.70 103.6	3.26 76.4	3.47 74.3	3.39 68.7	3.37 75.6	3.72, 3.86 60.2	
**E** →3)-β-D-Man*p*NAc-(1→	4.61 102.1	3.78 55.6	3.57 82.0	3.47 68.3	3.33 75.5	3.68, 3.75 61.3	1.99 175.1
**F** β-L-Rha*p*-(1→	4.43 102.3	3.33 71.3	3.55 72.7	3.81 68.9	4.48 68.0	1.20 16.5	
**F’** →3)-β-L-Rha*p*-(1→	-	3.34 71.3	3.70 79.5	3.91 72.8	4.50 68.1	1.17 16.4	

Spectra were obtained for ^2^H_2_O solutions at 25 °C, and acetone (δ_H_/δ_C_ 2.225/31.05 ppm) was used as an internal reference.

## Data Availability

All data that support the findings of this study are available on request from the corresponding author.

## References

[1] Scott M. (1968). The pathogenicity of *Aeromonas salmonicida* (Griffin) in sea and brackish waters. J. Gen. Microbiol..

[2] Dallaire-Dufresne S., Tanaka K.H., Trudel M.V., Lafaille A., Charette S.J. (2014). Virulence, genomic features, and plasticity of *Aeromonas salmonicida* subsp. salmonicida, the causative agent of fish furunculosis. Vet. Microbiol..

[3] Jimenez N., Lacasta A., Vilches S., Reyes M., Vazquez J., Aquillini E., Merino S., Regué M., Tomás J.M. (2009). Genetics and proteomics of *Aeromonas salmonicida* lipopolysaccharide core biosynthesis. J. Bacteriol..

[4] Merino S., de Mendoza E., Canals R., Tomás J.M. (2015). Functional genomics of the *Aeromonas salmonicida* lipopolysaccharide O-antigen and A-Layer from typical and atypical strains. Mar. Drugs.

[5] Merino S., Tomás J.M. (2016). The *Aeromonas salmonicida* lipopolysaccharide core from different subspecies: The unusual subsp. pectinolytica. Front. Microbiol..

[6] Wang Z., Vinogradov E., Larocque S., Harrison B.A., Li J., Altman E. (2005). Structural and serological characterization of the O-chain polysaccharide of *Aeromonas salmonicida* strains A449, 80204 and 80204-1. Carbohydr. Res..

[7] Wang Z., Liu X., Dacanay A., Harrison B.A., Fast M., Colquhoun D.J., Lund V., Brown L.L., Li J., Altman E. (2007). Carbohydrate analysis and serological classification of typical and atypical isolates of *Aeromonas salmonicida*: A rationale for the lipopolysaccharide-based classification of *A. salmonicida*. Fish Shellfish Immunol..

[8] Boltaña S., Tridico R., Teles M., Mackenzie S., Tort L. (2014). Lipopolysaccharides isolated from *Aeromonas salmonicida* and *Vibrio anguillarum* show quantitative but not qualitative differences in inflammatory outcome in *Sparus aurata* (Gilthead seabream). Fish Shellfish Immunol..

[9] Menanteau-Ledouble S., Kumar G., Saleh M., El- M. (2016). *Aeromonas salmonicida*: Updates on an old acquaintance. Dis. Aquat. Organ..

[10] Dalsgaard I., Nielsen B., Larsen J.L. (1994). Characterization of *Aeromonas salmonicida* subsp. salmonicida: A comparative study of strains of different geographic origin. J. Appl. Bacteriol..

[11] Baron S., Larvor E., Jouy E., Kempf I., Le Bouquin S., Chauvin C., Boitard P.M., Jamin M., Le Breton A., Thuillier B. (2021). Agreement between the categorization of isolates of *Aeromonas salmonicida* and *Yersinia ruckeri* by disc diffusion and MIC tests performed at 22 °C. J. Fish Dis..

[12] Kirkan S., Göksoy E.O., Kaya O. (2003). Isolation and antimicrobial susceptibility of *Aeromonas salmonicida* in rainbow trout (Oncorhynchus mykiss) in turkey hatchery farms. J. Vet. Med. B. Infect. Dis. Vet. Public Health..

[13] Rudel M.V., Vincent A.T., Attéré S.A., Labbé M., Derome N., Culley A.I., Charette S.J. (2016). Diversity of antibiotic-resistance genes in Canadian isolates of *Aeromonas salmonicida* subsp. salmonicida: Dominance of pSN254b and discovery of pAsa8. Sci. Rep..

[14] Poirel L., Jayol A., Nordmann P. (2017). Polymyxins: Antibacterial activity, susceptibility testing, and resistance mechanisms encoded by plasmids or chromosomes. Clin. Microbiol. Rev..

[15] Lamy B., Laurent F., Kodjo A. (2010). Validation of a partial rpoB gene sequence as a tool for phylogenetic identification of aeromonads isolated from environmental sources. Can. J. Microbiol..

[16] Yáñez M.A., Catalán V., Apráiz D., Figueras M.J., Martínez-Murcia A.J. (2003). Phylogenetic analysis of members of the genus *Aeromonas* based on gyrB gene sequences. Int. J. Syst. Evol. Microbiol..

[17] Miyata M., Inglis V., Aoki T. (1996). Rapid identification of *Aeromonas salmonicida* subspecies salmonicida by polymerase chain reaction. Aquaculture.

[18] Rivera I.G., Chowdhury M.A., Huq A., Jacobs D., Martins M.T., Colwell R.R. (1995). Enterobacterial repetitive intergenic consensus sequences and the PCR to generate fingerprints of genomic DNAs from *Vibrio cholerae* O1, O139, and non-O1 strains. Appl. Environ. Microbiol..

[19] Silley P. (2012). Susceptibility testing methods, resistance and breakpoints: What do these terms really mean?. Rev. Sci. Tech..

[20] Baron S., Granier S.A., Larvor E., Jouy E., Cineux M., Wilhelm A., Gassilloud B., Le Bouquin S., Kempf I., Chauvin C. (2017). Aeromonas diversity and antimicrobial susceptibility in freshwater-an attempt to set generic Epidemiological Cut-Off values. Front. Microbiol..

[21] Westpal O., Jann K. (1965). Bacterial lipopolysaccharides extraction with phenol-water and further applications of the procedure. Methods Carbohydr. Chem..

[22] Lee W., Tonelli M., Markley J.L. (2015). NMRFAM-SPARKY: Enhanced software for biomolecular NMR spectroscopy. Bioinformatics.

[23] Kaszowska M., Jachymek W., Niedziela T., Koj S., Kenne L., Lugowski C. (2013). The novel structure of the core oligosaccharide backbone of the lipopolysaccharide from the *Plesiomonas shigelloides* strain CNCTC 80/89 (serotype O13). Carbohydr. Res..

[24] Stojkovic K., Szijártó V., Kaszowska M., Niedziela T., Hartl K., Nagy G., Lukasiewicz J. (2017). Identification of D-galactan-III as part of the lipopolysaccharide of *Klebsiella pneumoniae* serotype O1. Front. Microbiol..

[25] Jachymek W., Niedziela T., Petersson C., Lugowski C., Czaja J., Kenne L. (1999). Structures of the O-specific polysaccharides from *Yokenella regensburgei* (*Koserella trabulsii*) strains PCM 2476, *2477*, 2478, and 2494: High-resolution magic-angle spinning NMR investigation of the O-specific polysaccharides in native lipopolysaccharides and directly on the surface of living bacteria. Biochemistry.

[26] Sigida E.N., Fedonenko Y.P., Shashkov A.S., Toukach P.V., Shelud’ko A.V., Zdorovenko E.L., Knirel Y.A., Konnova S.A. (2019). Structural studies of O-specific polysaccharide(s) and biological activity toward plants of the lipopolysaccharide from *Azospirillum brasilense* SR8. Int. J. Biol. Macromol..

[27] Sweeney M.T., Lubbers B.V., Schwarz S., Watts J.L. (2018). Applying definitions for multidrug resistance, extensive drug resistance and pandrug resistance to clinically significant livestock and companion animal bacterial pathogens. J. Antimicrob. Chemother..

[28] Ishiguro E.E., Ainsworth T., Shaw D.H., Kay W.W., Trust T.J. (1983). A lipopolysaccharide-specific bacteriophage for *Aeromonas salmonicida*. Can. J. Microbiol..

[29] Ishiguro E.E., Ainsworth T., Harkness R.E., Kay W.W., Trust T.J. (1984). A temperate bacteriophage specific for strains of *Aeromonas salmonicida* possessing A-layer, a cell surface virulence factor. Curr. Microbiol..

[30] Tomás J.M. (2012). The main *Aeromonas* pathogenic factors. ISRN Microbiol..

[31] Hofer R.N., Lin A., House B.C., Purvis C.N., Harris B.J., Symes S.J.K., Giles D.K. (2023). Exogenous polyunsaturated fatty acids (PUFAs) influence permeability, antimicrobial peptide resistance, biofilm formation and membrane phospholipid structure in an A-layer and non-A-layer strain of *Aeromonas salmonicida*. J. Fish Dis..

[32] Desriac F., El Harras A., Simon M., Bondon A., Brillet B., Le Chevalier P., Pugnière M., Got P., Destoumieux-Garzón D., Fleury Y. (2020). Alterins produced by oyster-associated *Pseudoalteromonas* are antibacterial cyclolipopeptides with LPS-Binding Activity. Mar. Drugs.

[33] Takiguchi S., Hirota-Takahata Y., Nishi T. (2022). Total synthesis and structural elucidation of ogipeptins. Org. Lett..

